# Disrupted TH17/Treg Balance in Patients with Chronic Low Back Pain

**DOI:** 10.1371/journal.pone.0104883

**Published:** 2014-08-14

**Authors:** Benjamin Luchting, Banafscheh Rachinger-Adam, Julia Zeitler, Lisa Egenberger, Patrick Möhnle, Simone Kreth, Shahnaz Christina Azad

**Affiliations:** Department of Anesthesiology and Pain Medicine, Ludwig-Maximilians University Munich, Munich, Germany; Toronto Western Hospital, Canada

## Abstract

Chronic low back pain (CLBP) is a leading cause of disability and costs in health care systems worldwide. Despite extensive research, the exact pathogenesis of CLBP, particularly the individual risk of chronification remains unclear. To investigate a possible role of the adaptive immune system in the pathophysiology of CLBP, we analyzed T cell related cytokine profiles, T cell related mRNA expression patterns and the distribution of T cell subsets in 37 patients suffering from nonspecific CLBP before and after multimodal therapy in comparison to 25 healthy controls. Serum patterns of marker cytokines were analyzed by Luminex technology, mRNA expression of cytokines and specific transcription factors was measured by real-time PCR, and distribution of TH1-, TH2-, TH17- and regulatory T cell (Tregs) subsets was determined by multicolor flow cytometry. We found that CLBP patients exhibit an increased number of anti-inflammatory Tregs, while pro-inflammatory TH17 cells are decreased, resulting in an altered TH17/Treg ratio. Accordingly, FoxP3 and TGF-β-mRNA expression was elevated, while expression of IL-23 was reduced. Serum cytokine analyses proved to be unsuitable to monitor the adaptive immune response in CLBP patients. We further show that even after successful therapy with lasting reduction of pain, T cell subset patterns remained altered after a follow-up period of 6 months. These findings suggest an involvement of TH17/Treg cells in the pathogenesis of CLBP and emphasize the importance of these cells in the crosstalk of pain and immune response.

**Trial Registration:**

German Clinical Trial Register: Registration Trial DRKS00005954.

## Introduction

Low back pain (LBP) is a common condition with a lifetime prevalence of nearly 84%. Although most patients recover completely within 4–8 weeks, a subset of patients is prone to develop chronic low back pain (CLBP). CLBP has become a major challenge for public health care systems worldwide [1]. The prevalence of CLBP is about 23%; around 12% of the afflicted patients are severely disabled [2], [3]. Still, mechanisms driving the chronification of low back pain syndromes remain largely elusive. Pathological physical conditions such as microtraumata, incorrect posture and degenerative processes as well as psychological factors such as overtaxing, emotional distress and inadequate coping have been described to contribute to the pathogenesis of CLBP [4], [5]. Increasing evidence indicates a pivotal role of the immune system in acute and chronic pain [6].

Recent studies have reported enhanced serum levels of pro-inflammatory cytokines in various pain syndromes [7], [8], [9], [10]. In the pathogenesis of CLBP, a possible impact of TNF-α was suggested [11]. Moreover, an increased expression of Il-17 in herniated and degenerated lumbar intervertebral discs has been reported, indicating a possible role of this cytokine in the chronification of pain [9].

While the innate immune system has been found to play an important role in acute pain [12], T-Lymphocytes as key players of the adaptive immune system are supposed to be of major importance [13], [14] in the pathogenesis of chronic pain. In patients with complex regional pain syndrome (CRPS) and in those suffering from abacterial chronic pelvic pain [15], [16], a TH1/TH2 imbalance with increased numbers of TH1 cells has been shown. In recent years, TH1/TH2 dichotomy has been expanded by two further CD4^+^ T cell lineages, Th17 and regulatory T cells (Tregs). These two T-cell subsets play prominent roles in immune functions: Th17 cells exerting pro-inflammatory effects are key players in the pathogenesis of autoimmune diseases and protection against bacterial infections, while Tregs function to restrain excessive effector T-cell responses. The role of both T cell subsets has extensively been analyzed in tumor growth and in the development of inflammatory and autoimmune diseases [17], [18], [19], [20], [21]. Recently published data also indicate an involvement of both T cell subsets in the development of chronic pain [22], [23], [24], [25]. For example, in patients with postherpetic neuralgia (PHN), increased Treg numbers have been found [26]. In addition, there is evidence that these cells play a central role in endogenous recovery from neuropathic pain [27]. Due to the antagonistic functions of TH17 and Treg cells, and in analogy to the well-known TH1/TH2 paradigm, the ratio between TH17 and Tregs is increasingly used to characterize immune responses.

In CLBP, however, specific alterations in the adaptive immune system have not conclusively been analyzed, yet.

In the current study, we investigated cytokine profiles and T helper cell subset compositions in CLBP patients and healthy controls. Our results indicate that CLBP is associated with characteristic alterations of T helper cell subsets: The TH17/Treg ratio was significantly decreased. We further provide evidence that these alterations persist even in those patients exhibiting significant pain reduction after participation in a standardized multimodal therapy program.

## Materials and Methods

### Ethics statement

The study followed the principles of the Declaration of Helsinki and was approved by the Ethics Committee of the LMU Munich.

### Subjects

During a prospective recruitment period of two years (September 2011 until September 2013), all patients seeking treatment for nonspecific CLBP at our pain clinic were assessed for study specific inclusion and exclusion criteria. Inclusion criteria were CLBP defined as low back pain persisting longer than two month, not attributable to a recognized specific pathological condition (e.g., disc herniation, any type of radiculopathy or other neuropathic pain, infection, tumor, osteoporosis, trauma, structural deformity or inflammatory disorders) and planned participation in a specific 4 week multimodal outpatient program (see Therapy). Exclusion criteria were concomitant autoimmune, chronic, inflammatory, neoplastic-, and psychiatric diseases, drug abuse and pregnancy as well as any preexisting long-term medication with opioids, non-opioid analgesics or co-analgesics. Healthy pain free volunteers without any signs or history of CLBP and concomitant diseases were asked for their participation in the study as controls. In total, 37 patients and 25 healthy controls matching the criteria listed above provided written informed consent and were enrolled in the study. None of the enrolled individuals had been treated with corticosteroids or had received immunomodulatory agents currently or in the past. Acute inflammatory diseases at the time of blood sampling were ruled out by measurement of body temperature and laboratory assessment of C-reactive-Protein (CRP) as wells as total- and differential leucocyte count. This study is registered on German Clinical Trial Register (Registration Trial DRKS00005954), but was not registered before enrollment of participants since all patients received only standard treatment and no further study-related interventions. The authors confirm that all ongoing and related trials for this drug/intervention are registered.

### Therapy

The multimodal outpatient program (MRIP, “Muenchner Ruecken Intensiv Programm”) performed at the University of Munich is a clinically established outpatient program for patients with chronic low back pain. In line with specific recommendations for the treatment of chronic disabling low back pain [2], [3], [28], the program follows a bio-psycho-social approach and comprises medical (examination, education), physical (exercise), work-related and behavioral therapy components. The program is conducted by specialists from at least four professional groups with different backgrounds (e.g. physicians, physiotherapists, psychotherapists, occupational therapists). The group size is limited to 10 patients. The duration of the program is 4 weeks, 5 days a week and 8 hours a day.

### Outcome assessment

Pain and stress levels were routinely evaluated by standardized questionnaires before treatment, at the end of the program and six months after completion of the program (follow-up). Patients were asked to rate their recalled average pain intensity using an 11-point numerical rating scale (NRS): 0 means “no pain” and 10 means “worst pain imaginable”. Self-perceived stress was evaluated using the Short Questionnaire on Current Burden (KAB, “Kurzfragebogen zur aktuellen Beanspruchung”). The KAB is able to repeatedly determine an individual’s psychological state under the conditions of acute or chronic stress and is highly sensitive to short-term or situational changes during a stressful experience [29]. The rating is based on a 6-point scale ranging from 1 to 6 for all six matched adjectives. Higher KAB values indicate increased perceived stress levels. Responders were defined as patients with improvements in NRS by ≥50% due to the treatment program. Healthy controls were asked to fill out questionnaires once.

### Blood sampling

Blood samples were taken from all patients before treatment, at the end of the program and at the follow-up six months after completion of the program. Blood samples from healthy volunteers were taken once at enrollment.

#### Cytokine Assessment

Blood samples were collected, centrifuged and stored in polypropylene aliquot tubes at −80°C. Samples were then assessed for levels of T cell related cytokines using a human cytokine multiplex immunoassay by Myriad Rules-Based Medicine Inc., Austin, Texas, USA. The multiplex microbead assay is based on Luminex technology and measures proteins in a similar manner to standard sandwich ELISA, with comparable sensitivity and range. Regarding the detection limits, the LLOQ (Lower Limit of Quantitation) for the cytokines were: TNF-α: 23.0 pg/ml, IFN-γ: 1.5 pg/ml, IL-4: 29.0 pg/ml, IL-6: 11.0 pg/ml, IL-10: 6.9 pg/ml, IL-17: 4.0 pg/ml, IL-23: 0.59 pg/ml. The LLOQ is the lowest concentration of an analyte in a sample that can be reliably detected and at which the total error meets the laboratory’s requirements for accuracy [30].

### Flow cytometric staining and analysis

After collection of heparinized venous blood samples, peripheral blood mononuclear cells (PBMCs) were separated by density gradient preparation over Ficoll-Uropoline (Sigma Aldrich, Taufkirchen, Germany). Hereupon, PBMCs were cryopreserved in RPMI freezing media containing 10% FCS and 10% DMSO [31] and stored at −30°C for 24 h and then at −196°C until measurement. After storage, samples were thawed rapidly in a water bath at 37°C and washed twice to eliminate DMSO. For TH1, TH2 and TH17 analysis, cells were stimulated 5 h with cell stimulation cocktail including protein transport inhibitors (Phorbol 12-myristate 13-acetate (PMA), ionomycin, brefeldin A and monensin, eBioscience, San Diego, CA, USA) according to the manufacturer’s protocol. Subsequently, cells were extracellulary stained with anti-human CD4 antibody and consecutively fixed and permeabilized (Fix-Perm-Solutions A and B, Life Technologies, Darmstadt, Germany) for intracellular staining with anti-human INF-γ (detection of TH1 cells), IL-4 (detection of TH2 cells) and IL-17 antibody (detection of TH17 cells, all Biolegend, San Diego, CA, USA). T cell distribution was measured by FACS analysis with the Attune Acoustic Focusing Cytometer (Life Technologies, Carlsbad, USA). Tregs were identified and quantified using multicolor flow cytometry after surface staining of PBMCs with mAbs specific for anti-human CD4, CD25 and CD127 and intracellular staining with an anti-human FoxP3 antibody. The frequencies of CD4^+^CD25^high^ and CD4^+^CD25^high^CD127^low^FoxP3^+^ T cells were expressed as percentage of total CD4^+^ T cells by sequential gating on lymphocytes. Isotype controls (Biolegend, San Diego, CA, USA) were given for compensation and confirmation of antibody specificity.

### RNA isolation and synthesis of cDNA

CD4^+^ cells were isolated from PBMCs by magnetic separation with Whole Blood CD4 MicroBeads (MACS Miltenyi Biotec, Auburn, CA, USA). Subsequently, total RNA was isolated using the mirVana miRNA Isolation Kit followed by a DNase-digest with Turbo DNA-free Kit (Ambion). Quantity and purity of the isolated RNA were measured using a NanoDrop ND-1000 spectrophotometer (Peqlab). After amplification of total RNA using TargetAmp 1-Round aRNA Amplification Kit (Epicentre Biotechnologies, Madison, WI, USA) and purification using RNeasy Mini Kit (Qiagen), cDNA synthesis was performed with SuperScript III First Strand Synthesis System (Invitrogen) and random hexamers (Qiagen).

### Quantitative real-time PCR (qPCR)

Quantitative RT-PCR was performed in duplicates with the LightCycler 480 instrument (Roche Diagnostics, Mannheim, Germany) using LightCycler 480 Probes Master and RealTime ready single assays (Roche Diagnostics) and UPL probes. The RealTime ready single assays contain target specific primers and a Universal ProbeLibrary LNA probe. Primer sequences and qPCR characteristics are given in Table 1. The cycling conditions comprised an initial denaturation phase at 95°C for 10 min, followed by 45 cycles at 95°C for 10 s, 60°C for 30 s and 72°C for 1 s. Relative mRNA expression was calculated by Relative Quantification Software (Roche Diagnostics) using an efficiency-corrected algorithm with standard curves and reference gene normalization against the reference genes succinate dehydrogenase complex subunit A (SDHA) and TATA box binding protein (TBP) as described in [32].

**Table 1:** RT-PCR Assay Characteristics and Primer Sequences.

Gene	Primer Sequence
SDHA	Roche RealTime Ready Single Assay ID 102136
TBP	Roche RealTime Ready Single Assay ID 101145
FoxP3	Roche RealTime Ready Single Assay ID 113503
IL-4	for 5′TGCCTCACATTGTCACTGC 3′
	rev 5′GCACATGCTAGCAGGAAGAAC 3′, UPL probe #38
IL-6	for 5′GATGAGTACAAAAGTCCTGATCCA 3′
	rev 5′CTGCAGCCACTGGTTCTGT 3′, UPL probe #40
IL-10	for 5′TGCCTTCAGCAGAGTGAAGA 3′
	rev 5′GCAACCCAGGTAACCCTTAAA 3′, UPL probe #67
IL-17	for 5′TGGGAAGACCTCATTGGTGT 3′
	rev 5′GGATTTCGTGGGATTGTGAT 3′, UPL probe #8
IL-23	for 5′CAGCTTCATGCCTCCCTACT 3′
	rev 5′GACTGAGGCTTGGAATCTGC 3′, UPL probe #14
TGF-β	for 5′ACTACTACGCCAAGGAGGTCAC 3′
	rev 5′TGCTTGAACTTGTCATAGATTTCG 3′, UPL probe #31
TNF-α	for 5′CAGCCTCTTCTCCTTCCTGAT 3′
	rev 5′GCCAGAGGGCTGATTAGAGA 3′, UPL probe #29
IFN-γ	for 5′GGCATTTTGAAGAATTGGAAAG 3′
	rev 5′TTTGGATGCTCTGGTCATCTT 3′, UPL probe #21
RoRγT	for 5′CAGCGCTCCAACATCTTCT 3′
	rev 5′CCACATCTCCCACATGGAC 3′, UPL probe #69

### Statistical analyses

All statistical analyses were performed using SigmaStat 12.0 (Systat Software, Chicago, USA). Every statistical analysis was started with testing for normal distribution using the Shapiro Wilk Test. Testing for differences between groups was accomplished by the T-Test for all data with normal distribution (IL-17, IL-23, TGF-β-mRNA, CD25^+^CD25^high^) and the nonparametric Mann-Whitney Rank Sum Test for all data without normal distribution (IL-6, IL-10, IL-23-mRNA, IFN-γ-mRNA, FoxP3-mRNA, RORγT-mRNA, Tregs, TH17 cells, TH17/Treg Ratio, TH1/TH2 Ratio). Values are expressed as mean ± standard deviation (SD) in the text and figures and p-values≤0.05 were considered statistically significant.

## Results

### Subjects and treatment variables

23 female and 14 male patients were enrolled. The median age of the patients at inclusion was 44.5 (range 21–73) years. The control group consisted of 25 (13 female/12 male) healthy pain free individuals aged 43.0 (range 24–54) years.

At inclusion, the average pain intensity of the patients was NRS 3.37 (±2.4) at rest and NRS 4.18 (±2.5) during movement. The average pain duration was 70.1 (±78.3) months. Using the KAB to evaluate the intensity of self-perceived stress, patients rated average KAB values at inclusion with 3.31 (±0.83). The average KAB of healthy controls was 1.80 (±0.64).

Upon therapy, 13 of 37 patients (35%) showed a significant reduction of pain scores (NRS) within 4 weeks, as defined by a decrease of pain ratings of ≥50%. They were therefore defined as therapy responders. Follow-up responders were defined as patients with persisting favorable effects according to the aforementioned criteria after 6 months.

### Serum cytokine profiles

**Figure 1:**
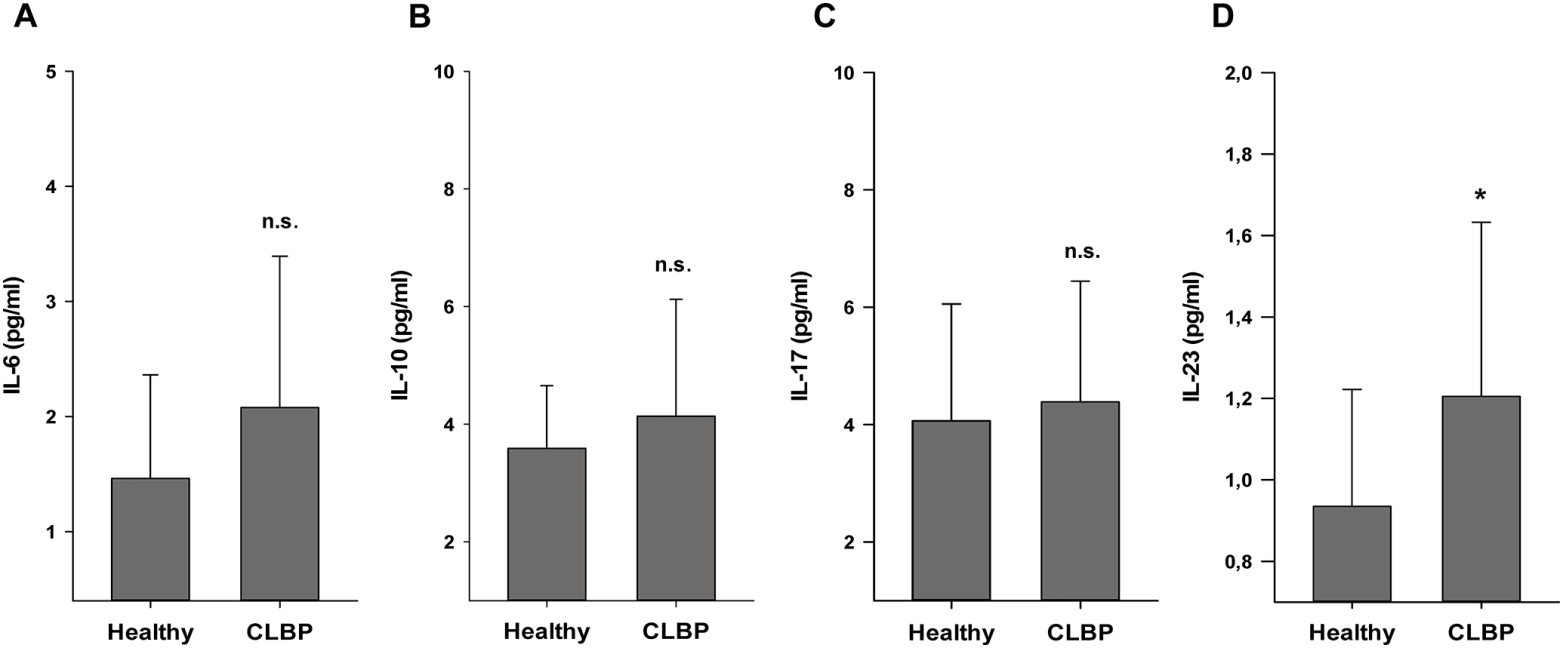
Concentrations of serum cytokines, determined by using a Human Cytokine multiplex immunoassay. No differences are found analyzing serum protein levels of IL-6, IL-10 and IL-17 between patients and healthy controls (Fig. 1A, 1B, 1C). Protein levels of pro-inflammatory cytokine IL-23 are significantly higher in the peripheral blood of patients with CLBP compared to healthy controls. Values are expressed as mean ± standard deviation. (IL-23: 0.94±0.29 pg/ml in healthy controls vs. 1.21±0.43 pg/ml in CLBP patients; p = 0.009; Fig. 1D).

Serum protein levels of TNF-α, IFN-γ and IL-4 were neither detectable in the peripheral blood of CLBP patients nor in blood samples of healthy controls. Generally, for both CLBP patients and healthy controls, the serum levels of IL-6, IL-10, Il-17 and IL-23 were found to be just marginally above the detection thresholds. No differences were found for IL-6, IL-10 and IL-17 (Figs. 1A, 1B, 1C). However, only levels of the pro-inflammatory cytokine IL-23 were found to be significantly higher in patients with CLBP before initiation of therapy as compared to healthy controls. (IL-23: 0.94±0.29 pg/ml in healthy controls vs. 1.21±0.43 pg/ml in CLBP patients; p = 0.009; Fig. 1D).

### mRNA expression of T cell cytokines

As measurements of specific cytokine profiles in serum turned out to be not conclusive, we determined the mRNA expression of cytokines and T cell specific transcription factors directly in the compartment of CD4^+^ cells of CLBP patients and healthy volunteers. By means of qPCR, we evaluated the mRNA expression of the TH1 cytokines TNF-α and IFN-γ, the TH2 cytokines IL-4 and IL-10, FoxP3 and TGF-β indicative for Tregs, and IL-6, IL-17, IL 23 and the transcription factor RORγT specific for TH17 cells.

**Figure 2:**
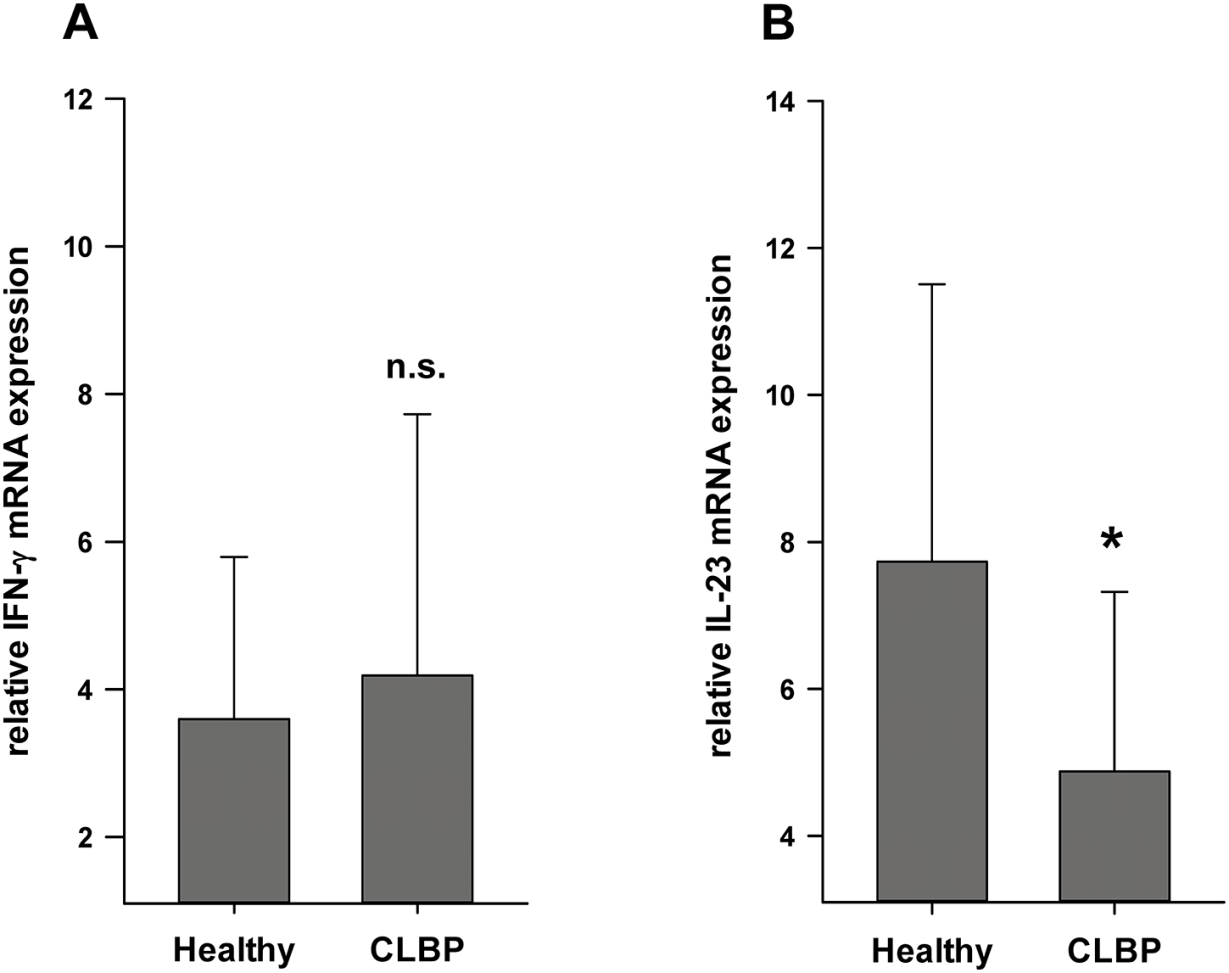
Expression levels of T cell related cytokine mRNA measured by qPCR. TNF-α, IL-4 and IL-10 were neither detectable in CD4^+^ T cells of CLBP patients nor in healthy controls. The expression of IFN-γ did not exhibit significant differences in CLBP patients as compared to healthy controls (IFN-γ: 4.19±3.54 in CLBP patients vs. 3.60±2.20 in healthy controls, n.s.; Fig. 2A) whereas IL-23 expression of in CD4^+^ T cell samples of CLBP patients was found to be significantly decreased (4.88±2.44 in CLBP patients vs. 7.73±3.77 in healthy controls, p = 0.006; Fig. 2B).

**Figure 3:**
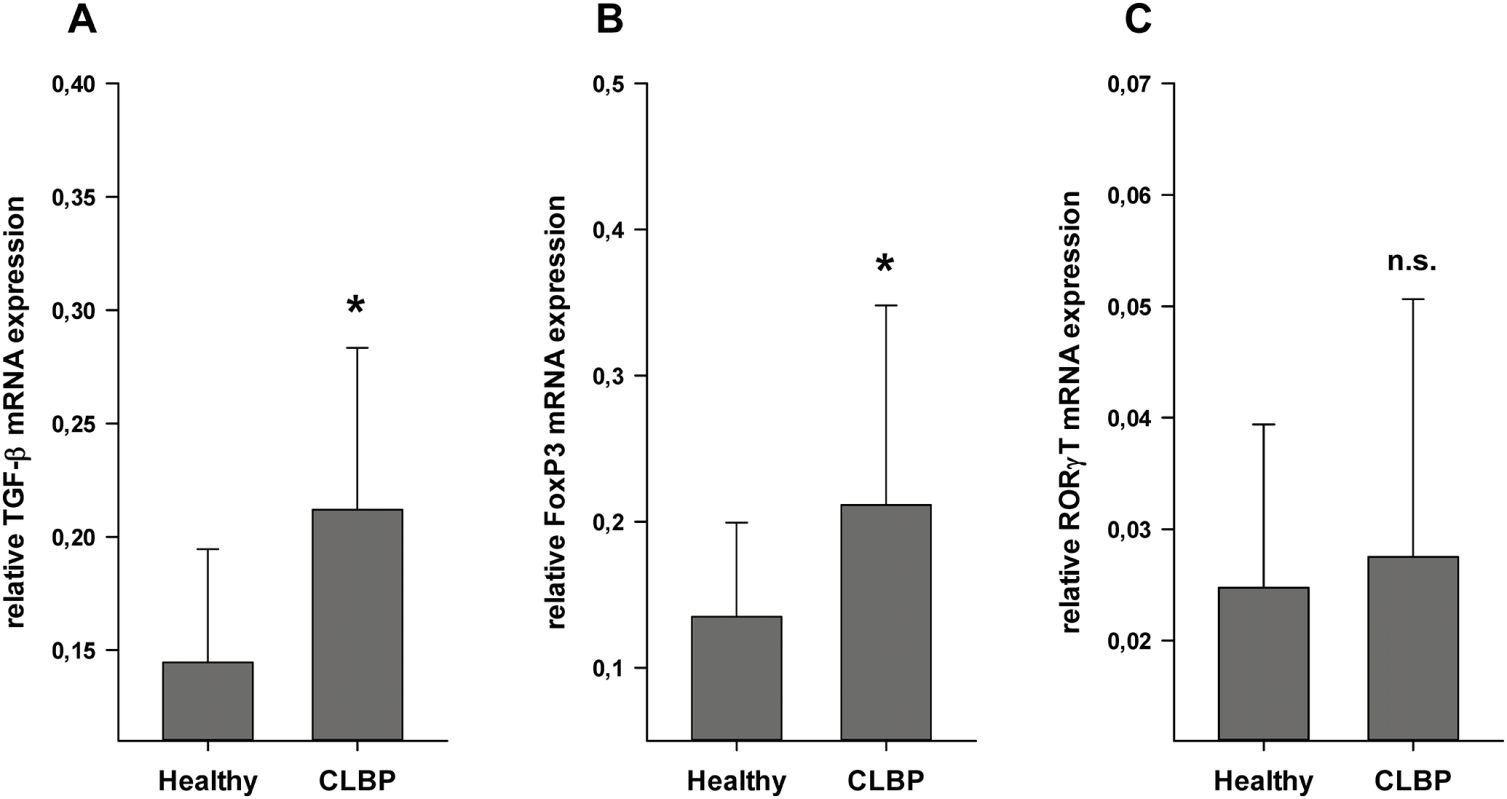
Expression levels of T cell subset related mRNA measured by qPCR. TGF-β and FoxP3 mRNA expression, specific for Tregs, was significantly higher in patients with CLBP than in healthy controls (TGF-β: 0.21±0.07 in CLBP patients vs. 0.14±0.05 in healthy controls, p = 0.014; Fig. 3A), (FoxP3: 0.21±0.14 in CLBP patients vs. 0.14±0.06 in healthy controls, p = 0.009; Fig. 3B). TH17 specific expression of IL-17 was neither detectable in CD4^+^ samples of CLBP patients nor in healthy controls. Expression levels of RORγT did not differ in CLBP patients and healthy controls (RORγT: 0.028±0.02 in CLBP patients vs. 0.025±0.01 in healthy controls, p = n.s.; Fig. 3C).

The expression of the TH1 specific cytokine IFN-γ did not exhibit significant differences in CLBP patients as compared to healthy controls (IFN-γ: 4.19±3.54 in CLBP patients vs. 3.60±2.20 in healthy controls, n.s., Fig. 2A). Expression levels of TNF-α, IL-4 and IL-10 were neither detectable in CD4^+^ T cells of CLBP patients nor in healthy controls. As shown in Fig. 2B, the expression of IL-23 in CD4^+^ T cell samples of CLBP patients was found to be significantly decreased compared to healthy controls (IL-23: 4.88±2.44 in CLBP patients vs. 7.73±3.77 in healthy controls, p = 0.006). The expression of both TGF-β and the transcription factor FoxP3 was significantly increased in CD4^+^ cells of CLBP patients compared to healthy controls, thereby implying an increased Treg abundance (TGF-β: 0.21±0.07 in CLBP patients vs. 0.14±0.05 in healthy controls, p = 0.014, Fig. 3A, FoxP3: 0.21±0.14 in CLBP patients vs. 0.14±0.06 in healthy controls, p = 0.009, Fig. 3B). Regarding factors specific for TH17 cells, the expression of IL-6 and IL-17 was neither detectable in CD4^+^ samples of CLBP patients nor in healthy controls. Expression levels of RORγT did not differ in CLBP patients and healthy controls (RORγT: 0.028±0.02 in CLBP patients vs. 0.025±0.01 in healthy controls, n.s., Fig. 3C). Taken together, qPCR results promoted the hypothesis that CLBP patients may exhibit altered distribution patterns of Treg and TH17 subsets whereas TH1/TH2 balance appeared to be unchanged.

### CLBP patients exhibit an increased Treg frequency while the TH17 frequency is decreased

**Figure 4:**
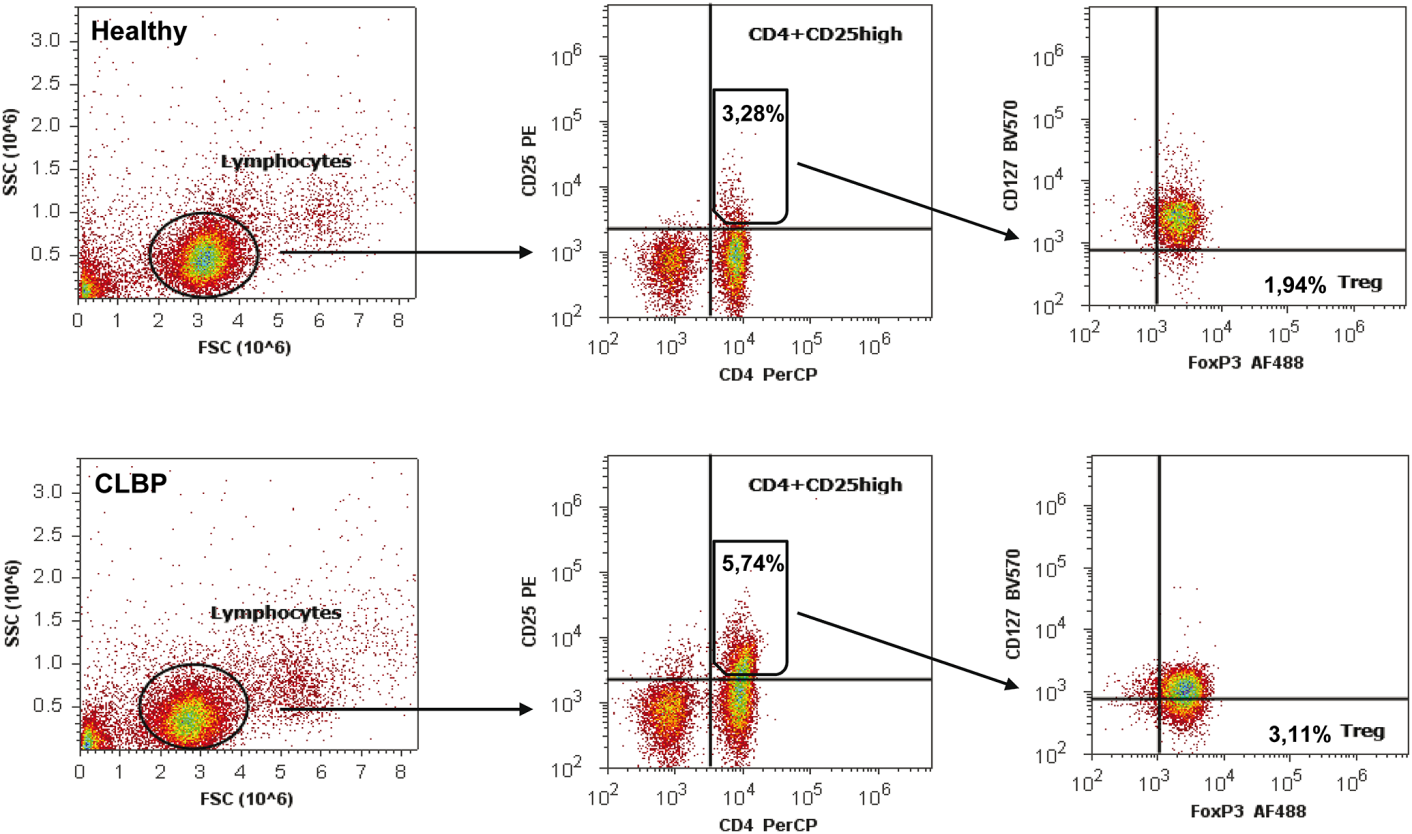
Gating strategy for the detection of Tregs. PBMCs extracellular stained with PerCP labeled anti-human CD4-antibody, PE labeled anti CD25-antibody, Brilliant Violet (BV570) labeled anti CD127-antibody and intracellular stained with Alexa Fluor (AF488) labeled anti-human FoxP3-antibody. Lymphocyte population was gated from PBMCs according to forward scatter (FSC) characteristics and side scatter (SSC) characteristics (left). Gated lymphocytes were then separated in CD4^+^CD25^high^ cells/T cells (middle) and CD4^+^CD25^high^CD127^low^FOXP3^+^ cells/CD4^+^T cells (right, named Treg). Upper row represents the result of a healthy control with less CD4^+^CD25^high^ T cells (3.28%) and less CD4^+^CD25^high^CD127^low^FoxP3^+^ T cells (1.94%) compared to a patient with CLBP (lower row, 5.74% and 3.11%).

**Figure 5:**
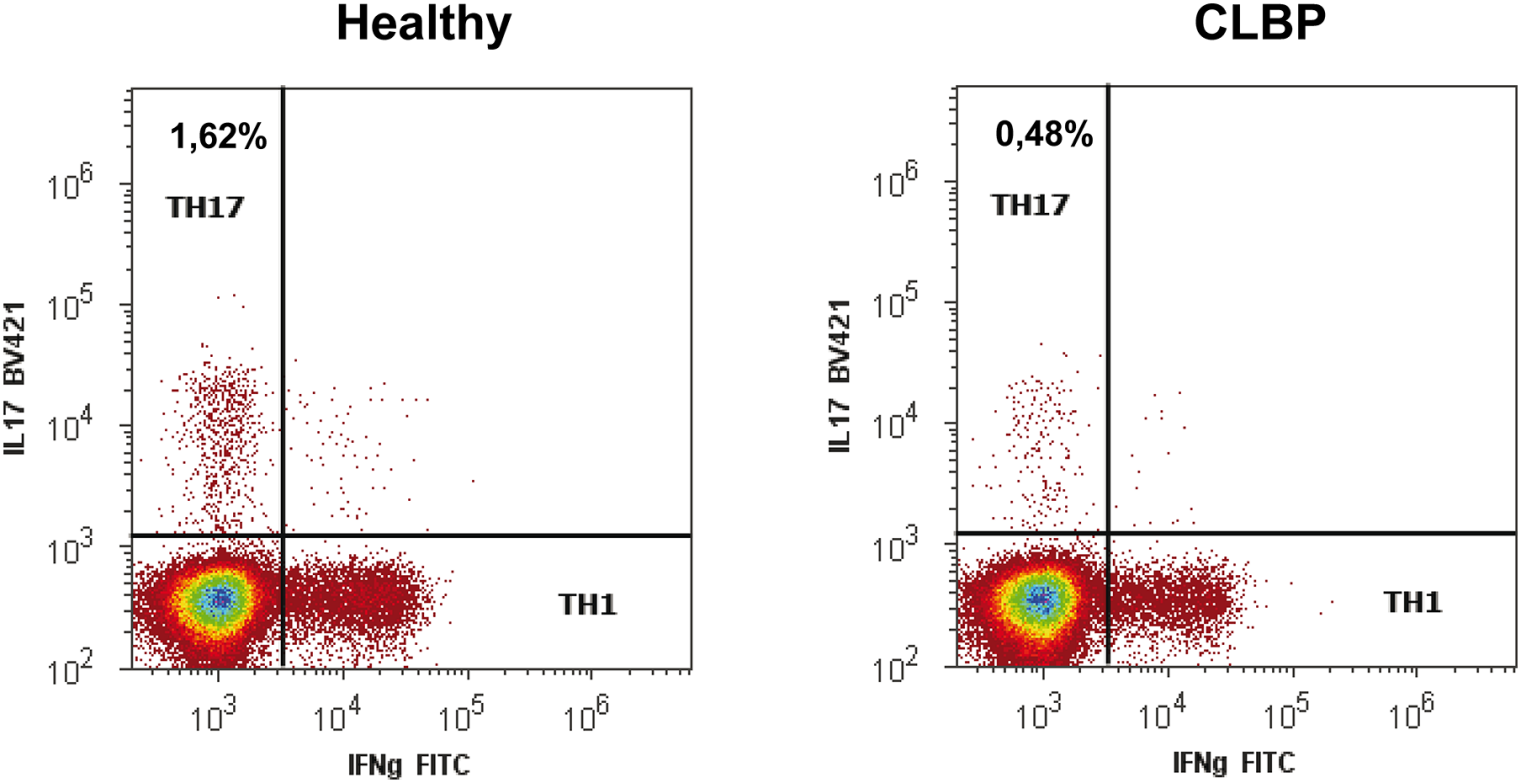
Gating strategy for the detection of TH1 and TH17 cells. PBMCs stimulated with cell stimulation cocktail for 5 h followed by intracellular staining with Brilliant Violet (BV421) labeled anti-human IL-17 antibody and FITC labeled anti-human IFN-γ antibody.

To test this hypothesis, we next evaluated the distribution of T cell subsets in blood samples of patients and healthy volunteers by flow cytometric analyses. The relative number of Tregs was assessed by using two different staining protocols: First, with antibodies specific for CD4^+^CD25^high^ cells and second, specific for CD4^+^CD25^high^CD127^low^FoxP3^+^ cells (Gating strategy is displayed on Fig. 4). TH17 cells were identified by intracellular staining with anti-human IL-17 antibody (gating strategy is displayed on Fig. 5).

**Figure 6:**
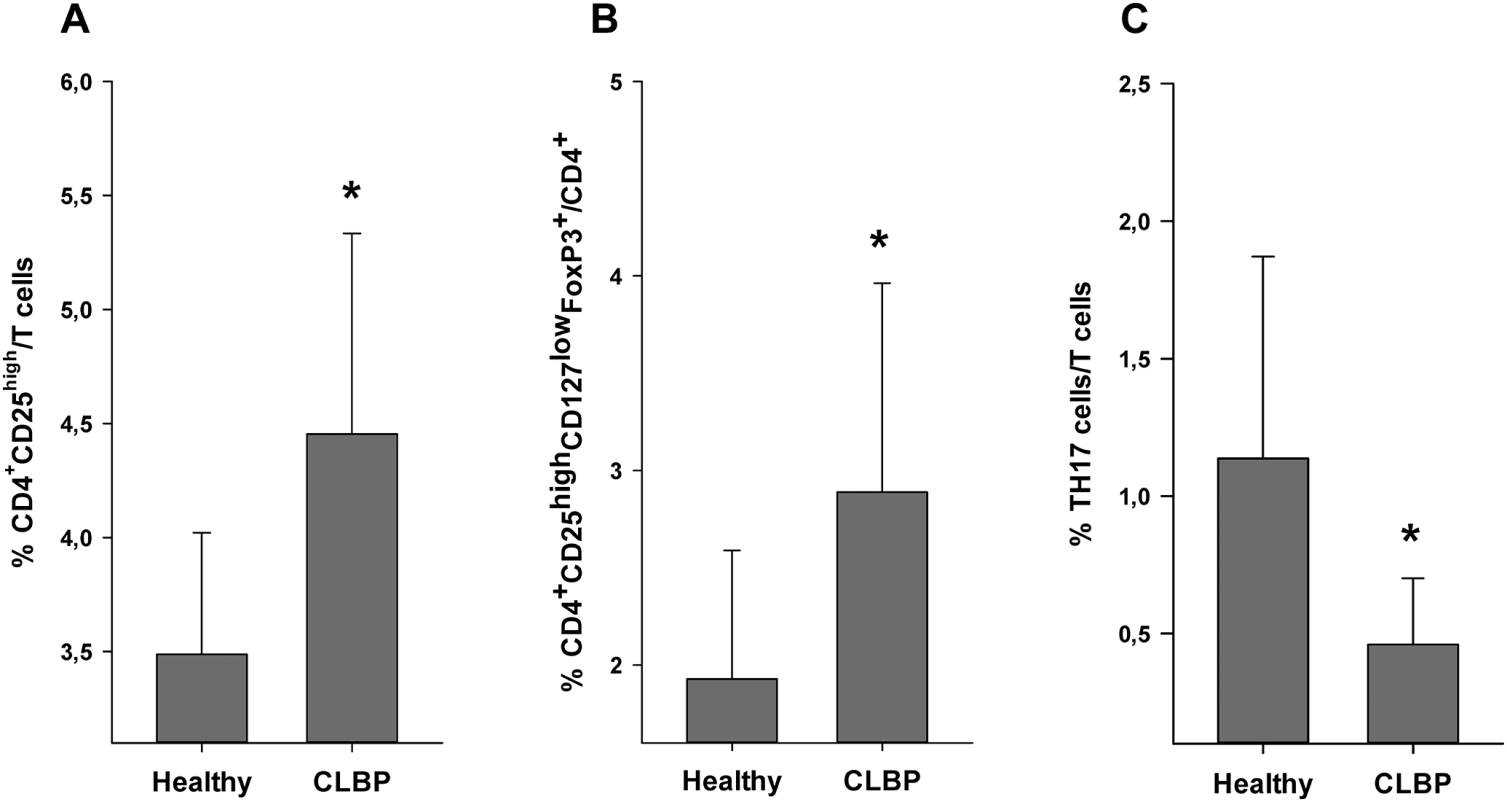
Flow cytometric quantification of Tregs and TH17 cells. Results show significantly higher percentage of anti-inflammatory Tregs in patients with CLBP in both staining protocols (CD4^+^CD25^high^ cells: 4.45±0.88% in CLBP patients vs. 3.49±0.53% in healthy controls, p<0.001; Fig. 6A), (CD4^+^CD25^high^CD127^low^FoxP3^+^ cells: 2.89±1.07% in CLBP patients vs. 1.93±0.66% in healthy controls, p = 0.001; Fig. 6B). Number of TH17 cells as percentage of T cells in peripheral blood show significantly lower percentage of pro-inflammatory TH17 cells in patients with CLBP (TH17 cells: 0.46±0.24% in CLBP patients vs. 1.14±0.73% in healthy controls, p<0.001; Fig. 6C).

**Figure 7:**
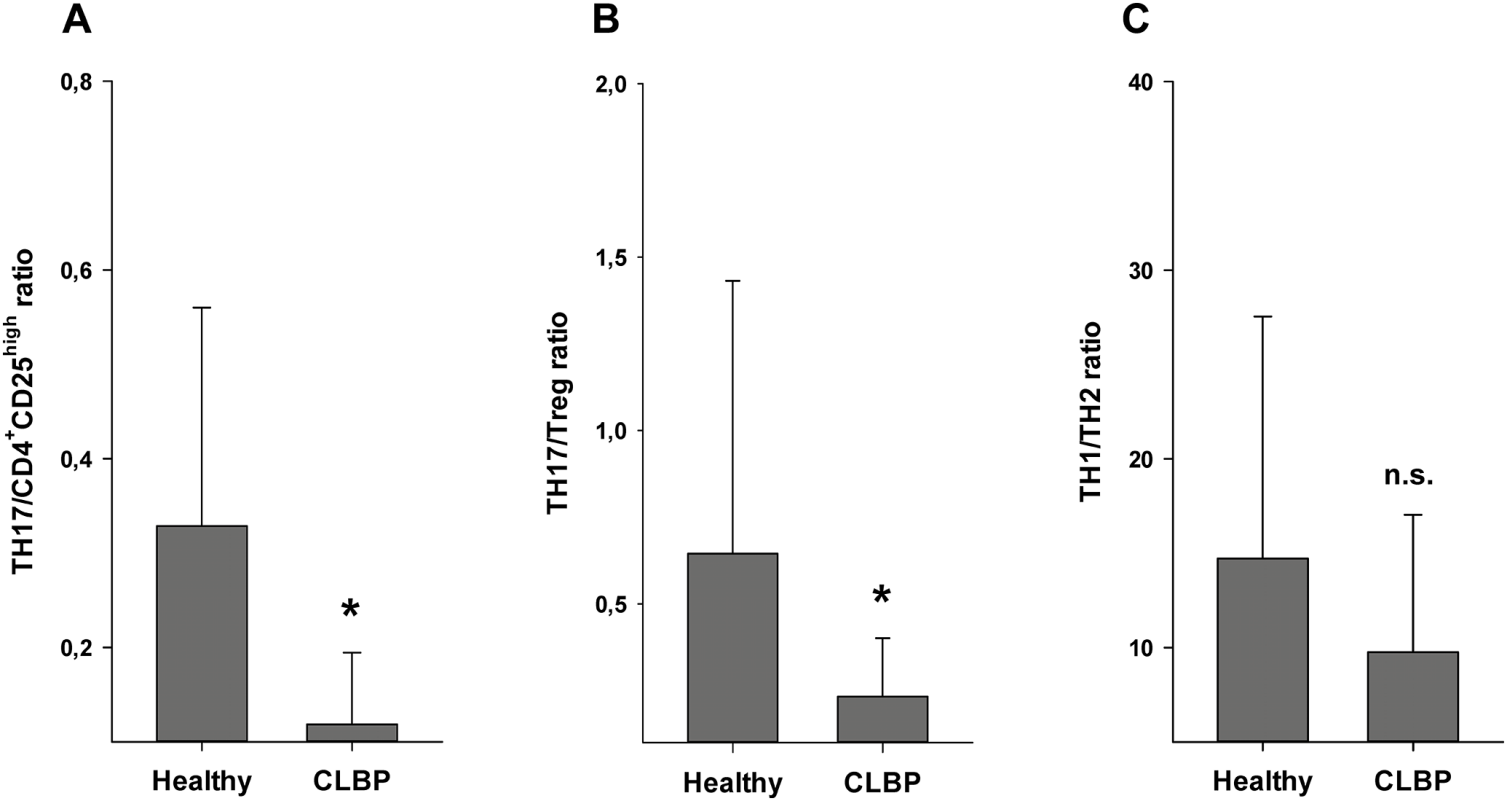
Ratios of TH17/CD4^+^CD25^high^, TH17/Tregs and TH1/TH2 cells. Ratios of Th17/CD4^+^CD25^high^ and Th17/CD4^+^CD25^high^CD127^low^FoxP3^+^ were significantly decreased in CLBP patients as compared to healthy controls (Th17/CD4^+^CD25^high^: 0.12±0.08 in CLBP patients vs. 0.33±0.23% in healthy controls, p<0.001; Fig. 7A), (Th17/CD4^+^CD25^high^CD127^low^FoxP3^+^: 0.23±0.17 in CLBP patients vs. 0.64±0.79 in healthy controls, p<0.001; Fig. 7B). Ratio of TH1/TH2 cells in peripheral blood of patients with CLBP and healthy controls were tendencially decreased in patients with CLBP, but did not reach significance (9.76±7.27 in CLBP patients vs. 14.72±12.81 in healthy controls, p = 0.19, Fig. 7C).

With both Treg staining protocols, a significantly increased frequency of Tregs was seen in CLBP patients as compared to healthy controls. FACS analysis applying the CD4^+^CD25^high^ staining protocol resulted in 4.45±0.88% CD4^+^CD25^high^ cells in CLBP patients vs. 3.49±0.5%, CD4^+^CD25^high^ cells in healthy controls (p<0.001, Figure 6A). CD4^+^CD25^high^CD127^low^FoxP3^+^ staining as a more specific staining protocol for Tregs revealed similar results with 2.89±1.07% Tregs in CLBP patients vs. 1.93±0.66% Tregs in healthy controls (p = 0.001, Figure 6B). The frequency of TH17 cells, however, was found to be significantly decreased in CLBP patients as compared to healthy volunteers (TH17: 0.46±0.24% in CLBP patients vs. 1.14±0.73% in healthy controls, p = <0.001, Figure 6C). Conclusively, ratios of Th17/CD4^+^CD25^high^ resp. Th17/CD4^+^CD25^high^CD127^low^FoxP3^+^ were significantly decreased in CLBP patients as compared to healthy controls (Th17/CD4^+^CD25^high^: 0.12±0.08 in CLBP patients vs. 0.33±0.23 in healthy controls, p<0.001, Fig. 7A; Th17/CD4^+^CD25^high^CD127^low^FoxP3^+^: 0.23±0.17 in CLBP patients vs. 0.64±0.79 in healthy controls, p<0.001, Fig. 7B).

### TH1/TH2 balance is not altered in CLBP patients

As depicted in Fig. 7C, TH1/TH2 balance did not reveal significant differences between CLBP patients and healthy controls; however, a trend towards a decreased TH1/TH2 ratio was observed (TH1/TH2: 9.76±7.27 in CLBP patients vs. 14.72±12.81 in healthy controls, p = n.s.).

### T cell ratios remain altered in CLBP patients after multimodal therapy

**Figure 8:**
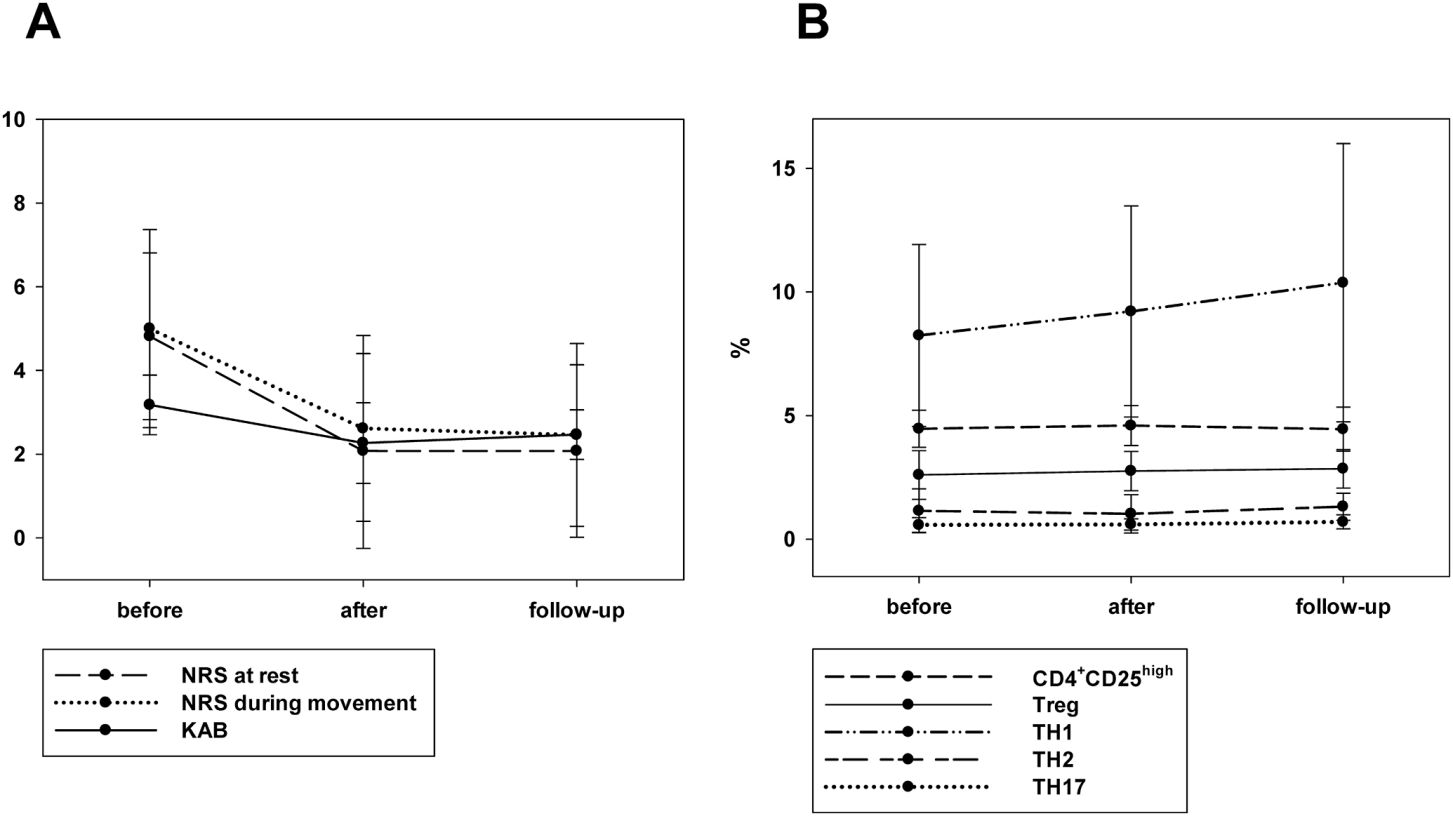
NRS pain scores, KAB stress scores and T cell subsets before and after treatment. 35% [n = 13] of all patients benefited by the 4 weeks intensive multimodal therapy with long lasting pain- and stress reduction (Fig. 8A). Even all responders showed a significant pain- and stress reduction of ≥50%, no transformation were observed regarding T cell subsets. None of our analyzed T cell subsets (TH1, TH2, TH17, Tregs) normalized after successful therapy (Fig. 8B). (NRS at rest before/after: p = 0.025, NRS at rest before/follow up: p = 0.003, NRS during movement before/after: p = 0.046, NRS during movement before/follow up: p = 0.012, KAB before/after: p = 0.024, KAB before/follow up: p = 0.019).

To evaluate the impact of therapeutic interventions on the observed T cell subset alterations in CLBP patients, the distribution of TH cells subsets (TH1, TH2, TH17 and Tregs) was analyzed in the group of therapy responders before, immediately after therapy and 6 months later. As depicted in Fig. 8A, these patients showed an ongoing decrease of NRS by ≥50% due to the treatment program. The pain reduction was also accompanied by a decrease of the KAB values. However, as shown in Figure 8B, this therapeutic effect was not reflected in any respective adaptation of the T cell subsets.

## Discussion

Pathomechanisms driving the chronification of low back pain still remain largely elusive. While a growing body of evidence suggests a pivotal role of adaptive immune responses in the pathogenesis of chronic pain, these issues have not conclusively been analyzed for CLBP, yet.

Our results indicate that CLBP is associated with characteristic alterations of T helper cell subsets: The ratio between regulatory T cells, playing a vital role in controlling adaptive immune responses, and TH17 cells, one of the key effector T cells mediating autoimmunity [33], was significantly decreased. We further provide evidence that these alterations persist even in these patients exhibiting significant pain reduction after participation in a standardized multimodal therapy program [3].

Assuming that cytokines as central mediators of cellular immunity may mirror immune cell functions, we first analyzed seven T cell related cytokines (TNF-α, IFN-γ, IL-4, IL-6, IL-10, IL-17, IL-23) in serum samples of CLBP patients and healthy controls. TNF-α, IFN-γ and IL-4 were below the detection limits in patients as well as in healthy controls, and the results of the remaining four analytes were only slightly above the detectable limit. Values for the proinflammatory cytokines IL-6 and IL-17 in blood samples of CLPB patients were slightly elevated as compared to controls. However, this finding may be of limited clinical relevance as normal plasma concentrations for IL-6 of healthy controls are about 1 pg/ml with immense increases in situations of severe systemic infection ranging up to 10.000-fold. Our results demonstrate only an 1.5-fold increase in IL-6 levels in patients with CLBP, which could even occur after physical activity or in obesity [34]. However, the relevance of cytokine measurements should generally be regarded with caution as serum levels of most cytokines are influenced by a complex interplay of macrophages/monocytes, fibroblasts, endothelial−/epithelial cells and dendritic cells thus complicating the extrapolation from plasma cytokines to immune cell functions. Moreover, ranges of detection exhibits considerable variances between the different assays used [35]. Even different types of Luminex-based platforms exhibit differences in their ability to measure serum levels of cytokines and thus, may be more useful in studies in which relative rather than absolute changes in cytokines are of interest [36], [37].

Overall, these data suggest that serum levels of cytokines are not suitable to monitor the adaptive immune response in CLBP and prompted us to analyze the expression of cytokines directly in the compartment of CD4^+^ cells as central players of the T cell response. While no differences in the expression of TH1 and TH2 cytokines were observed, qPCR results clearly pointed to an increased abundance of Tregs in CLBP patients, as expression of both TGF-β and the transcription factor FoxP3 were significantly increased. Moreover, expression of IL-23 was clearly decreased supporting the assumption that TH17 frequency may be reduced. IL-17 and RORγT, however, did not differ significantly between CLBP and controls which may be due to the fact that the subset of TH17 cells per se is only less than 2% of CD4+ cells. Thus, resolving differences of cytokine expression without prior cell sorting may be difficult. The opposite results of increased IL-23 protein levels and decreased IL23-mRNA-expression is in line with a wide body of literature showing a big discrepancy between mRNA expression and protein levels as a result of control mechanisms. These can affect post-transcriptional, translational and protein degrading processes [38], [39].

Our findings encouraged us to investigate T cell subset compositions by flow cytometric analyzes. We used standard staining procedures to identify TH1, TH2, and TH17 cells, whereas for identification of anti-inflammatory Tregs, both classic extracellular staining of CD4^+^CD25^high^ and a more specific extra- and intracellular staining of CD4^+^CD25^high^CD127^low^FoxP3^+^ was applied. As activated human T cells can transiently express FoxP3 and CD25, differentiation of Tregs from activated effector T cells by only using these two markers may suffer from inaccuracies. CD127 is a newly described surface marker that allows distinguishing regulatory T cells from other CD25^+^ cells [40]. For TH17 identification, we chose two experimental approaches: determination the mRNA expression of the TH17 specific transcription factor RORγT by qPCR, and FACS analyses of IL-17 production, which has revealed as a very reliable method to identify TH17 cells [41]. However, flow cytometry staining protocols combining IL-17 with further markers, e.g. CD161 or CCR6, may further refine these measurements and thus may be implemented in future studies.

Flow cytometry clearly proved the assumed alterations of the TH17/Treg balance, as a significantly increased frequency of Tregs and decreased frequency of TH17 cells was observed in our CLBP patients. Even in flow cytometric analyzes, no differences in the TH1/TH2 ratio were detectable. There are several investigations which point to a beneficial role of anti-inflammatory cells and cytokines together with a detrimental function of a pro-inflammatory immune response in pain patients [6], [7], [8], [9]. In contrast to these findings, our results showing an anti-inflammatory shift on cellular level are in accordance with other chronic diseases like mild depression or chronic fatigue syndrome [42], [43]. A potential explanation for our findings on TH17/Treg balance may therefore be that pain-related, long lasting chronic stress and fatigue induces an ongoing dysregulation of immune cells towards an anti-inflammatory phenotype [44], [45], [46]. On the other hand, it may also be discussed that dysregulation of the TH17/Treg balance may exist first, thus predisposing the affected individuals to experience chronification of pain symptoms. The latter theory may be supported by our surprising findings that the observed TH17/Treg imbalance persisted despite clinical improvement after multimodal therapy even after a follow-up period of 6 months.

In summary, we found a persisting TH17/Treg imbalance with an increased count of anti-inflammatory Tregs and a decreased number of pro-inflammatory TH17 cells in peripheral blood of CLBP patients pointing to a strong association between chronic pain and immune suppression rather than immune activation. Importantly, these findings are not reflected by serum cytokine concentration, indicating a major role of specific T cell subset measurements in the analysis of pain-related immune responses.

Taken together, the results of the current study suggest an involvement of TH17/Treg in the pathogenesis of CLBP and emphasize the importance of these cells in the crosstalk of pain and immune response.
